# Function of Nr4a Orphan Nuclear Receptors in Proliferation, Apoptosis and Fuel Utilization Across Tissues

**DOI:** 10.3390/cells8111373

**Published:** 2019-11-01

**Authors:** Jacob A. Herring, Weston S. Elison, Jeffery S. Tessem

**Affiliations:** 1 Nutrition, Dietetics and Food Science Department, Brigham Young University, Provo, UT 84602, USA; herrin06@gmail.com (J.A.H.); weston.elison@gmail.com (W.S.E.); 2 Microbiology and Molecular Biology Department, Brigham Young University, Provo, UT 84602, USA

**Keywords:** Nr4a1, Nr4a2, Nr4a3, nuclear hormone receptors, metabolism, fuel utilization, proliferation, apoptosis

## Abstract

The Nr4a family of nuclear hormone receptors is composed of three members—Nr4a1/Nur77, Nr4a2/Nurr1 and Nr4a3/Nor1. While currently defined as ligandless, these transcription factors have been shown to regulate varied processes across a host of tissues. Of particular interest, the Nr4a family impinge, in a tissue dependent fashion, on cellular proliferation, apoptosis and fuel utilization. The regulation of these processes occurs through both nuclear and non-genomic pathways. The purpose of this review is to provide a balanced perspective of the tissue specific and Nr4a family member specific, effects on cellular proliferation, apoptosis and fuel utilization.

## 1. Introduction

The Nr4a family consists of three members, Nr4a1 (Nur77/TR3/NGFI-B), Nr4a2/Nurr1 (TINUR/NOT) and Nr4a3/NOR1 (MINOR/CSMF). The three members have a high degree of sequence homology, with each containing a ligand-independent activation function-1 domain, transactivation domain necessary for transcriptional activity and cofactor binding, a DNA binding domain and a ligand binding domain containing a ligand-dependent AF-2 transactivation domain [1,2,3]. While currently defined as orphaned receptors with no known endogenous ligand, recent reports suggest that the Nr4a family’s transcription factor function may be regulated through binding unsaturated fatty acids in the ligand binding domain [4,5,6].

The Nr4a family binds directly as monomers or homodimers to promoters of target genes that contain the NBRE (NGFIB Response Element-AAAGGTCA) motif [7]. The Nr4as can also form heterodimers and bind to the NuRE (Nur Response element-AAT(G/A)(C/T)CA) [8,9]. Finally, Nr4a1 and Nr4a2 have been shown to forms dimers with retinoid X receptors and bind to the DR5 elements [10,11]. While there is a high degree of similarity between the three family members, the different members have differing affinity for co-factors and response elements, thus giving specificity to each [12]. 

The Nr4a family is widely expressed across various tissues [13]. The family was first discovered in the nervous system [14,15,16,17]. Nr4a’s have been shown to be critical in the hematopoietic system [18], adipose tissue [19], liver [20], muscle [21] and β-cells [22], among other tissues [23,24]. In these tissues, the function of Nr4a family members fall into one of two categories. The majority of Nr4a activity is due to direct activation or repression of transcriptional target expression. Interestingly, a growing body of information is demonstrating a direct non-genomic role of Nr4a’s through interaction with binding partners [25]. 

While the Nr4a’s are associated with various cellular processes, three are of particular interest. The Nr4a’s, across various tissue types, impinge on cellular proliferation, apoptosis and fuel utilization. These pathways are intimately connected. The cells ability and choice of macronutrients as a fuel source and their ability to utilize these molecules, greatly affects proliferation and cell survival. The type of metabolic pathways and choice of fuel oxidation is tightly coordinated with a cells ability to proliferate. Similarly, defective fuel utilization pathways or lack of oxidizable macronutrients, can impinges on apoptosis. Finally, signals that induce cell proliferation and apoptosis are in direct opposition to each other. This review will focus on the genomic and non-genomic Nr4a targets that allow for modulation of proliferation, apoptosis and fuel utilization. 

## 2. Proliferation

The Nr4a subfamily is known to regulate cellular proliferation in a tissue dependent manner. Key genes shown to be regulated by Nr4a’s include cyclins, cyclin dependent kinases and other ancillary cell cycle genes. Due to this, Nr4a1, Nr4a2 and Nr4a3 are potential therapeutic targets in many cancers, however their specific roles vary between tissues and among tumors from the same organ. The genes that have been shown to be transcriptionally regulated by the Nr4a’s that are essential for cellular proliferation are summarized in Table 1.

### 2.1. Liver

Liver tissue has a remarkable ability to regenerate. Controlled cell proliferation is key in this process to regenerate tissue without becoming malignant. Hepatic stellate cell proliferation has also been identified as an issue in liver fibrosis. In both situations the Nr4a subfamily is a regulatory factor.

#### 2.1.1. Anti-Proliferation

When subjected to partial hepatectomy Nr4a1 deficient mice generated by homologous recombination showed increase in NF-κB and STAT3 protein levels which in turn induced Cyclin B1, Cyclin D1, Cyclin E1, Cdk4 and Cdk2 expression within 24 h, demonstrating that Nr4a1 limits regeneration in hepatocytes [26]. It is important to note, however, that recent findings have shown that the Nr4a1 knock out mouse used in this study (and others) expresses the N-terminal domain of Nr4a1 and that this is sufficient to bind and stabilize HIF1α [52]. While no change in hepatocyte proliferation was demonstrated in this study, it does emphasize the need to validate these results in the floxed Nr4a1 model. In the hepatic stellate cell HSC-T6 cell line Nr4a2 inhibition by siRNA led to increased cell proliferation and decreased phosphorylation of the MAPK pathway members ERK1/2, p38 and JNK [28]. This result was supported by a marked increase in ERK1/2 and p38 phosphorylation observed in HSC-T6 cells overexpressing Nr4a2, along with decreased proliferation [29]. 

#### 2.1.2. Pro-Proliferation

While Nr4a1 and Nr4a2 inhibit proliferation in liver tissues, Nr4a3 promotes proliferation in both hepatocytes and hepatic stellate cells. Mice treated with Nr4a3 shRNA were subjected to partial hepatectomy. Decreased expression of Cyclin D1, Cyclin E1, VCAM1 and PCNA were observed, as well as lower liver weight. Overexpression of Nr4a3 increased expression of these same genes. Chromatin immunoprecipitation (ChIP) revealed that Nr4a3 directly interacts with the Cyclin D1 promoter, demonstrating that it is a direct Nr4a3 target in hepatocytes [27]. In hepatic stellate cells TGF-β1 was sufficient to drive activation and proliferation. TGF-β1 was observed to increase expression of Nr4a3 and Nr4a3 inhibition impaired primary hepatic stellate cell proliferation, demonstrating Nr4a3′s role in TGF-β1 mediated hepatic stellate cell activation and proliferation [53]. As observed with hepatocytes, the Nr4a subfamily has opposing roles in controlling proliferation which may assist in balancing healthy benign growth. 

### 2.2. Muscle

In smooth and cardiac muscle, proliferation is often associated with hypertrophy and disease pathogenesis such as atherosclerosis, restenosis and heart disease. Again, we see Nr4a’s opposing roles in regulating this process.

#### 2.2.1. Anti-Proliferation

Vascular smooth muscle cells (VSMCs) have been used to study the Nr4a role in smooth muscle. VSMCs derived from pulmonary artery and saphenous vein, PDGF, 5-HT or stress were shown to cause proliferation. When Nr4a1 was overexpressed, it inhibited proliferation by blocking the STAT3/Pim-1/NFAT pathway members which decreased Cyclin D1 and PCNA expression [30,31,32]. Nr4a1 knock down increased stress induced proliferation and VSMCs from Nr4a1 deficient mice had increased proliferation [31,32]. Nr4a1 expression was increased in VSMCs treated with LDL-activated macrophages. Nr4a1 overexpression lead to lower proliferation rates, less tunica intima growth and induction of p27^Kip1^, all of which ultimately blocked VSMC proliferation induced vessel narrowing [33]. In cardiomyocytes, Nr4a1 was found to block proliferation induced by neuropeptide Y which has been linked to cardiac hypertrophy [54].

Nr4a2 has a similar affect in neonatal tunica intima VSMCs as Nr4a1 does in other VSMCs. Nr4a2 knock down increased proliferation, while overexpression decreased proliferation by increasing p27^Kip1^ expression [34]. Nr4a2 also inhibits proliferation in cardiac myocytes. Isoproterenol has been found to increase cardiomyocyte proliferation and when Nr4a2 was overexpressed isoproterenol stimulated proliferation was decreased. The decreased proliferation was due to Nr4a2 mediated increases in DUSP2 and DUSP14 expression which decreased ERK1/2 and AKT phosphorylation [38]. Together, Nr4a1 and Nr4a2 protect against cardiac hypertrophy and vessel narrowing due to stress and other causes such as atherosclerosis.

#### 2.2.2. Pro-Proliferation

Nr4a3 again plays a counter role to Nr4a1 and Nr4a2 in VSMCs. PDGF and hypoxia induce Nr4a3 in VSMCs [35,36]. Nr4a3 promotes proliferation by inducing Cyclin D1, Cyclin D2 and PCNA, which effect was lost with siNr4a3 treatments [35,36,37]. While Nr4a1 and Nr4a2 are protective against hypertrophy and excess muscle tissue growth, Nr4a3 promotes proliferation. This proliferation may be beneficial in situations but in COPD induced hypoxia it is implicated in causing pulmonary hypertension [36],

### 2.3. β-Cell

#### Pro-Proliferation

Nr4a1 and Nr4a3 are both pro-proliferative in the β-cell. As β-cell loss and dysfunction are key aspects of diabetes pathology, β-cell proliferation would enable islet transplants and restored insulin secretion. Nkx6.1, a key β-cell transcription factor upregulates Nr4a1 and Nr4a3 expression. Nr4a1 and Nr4a3 promote proliferation by increasing E2F1 which increases Cyclin E1 concentrations as well as by increasing Ube2c levels which degrades p21^Cip1^ [22]. Cdk5r1 expression was also increased and is necessary for Nr4a1 and Nr4a3 driven β-cell proliferation, by increasing pRb phosphorylation and allowing E2F1 mediated increases in cell cycle gene expression [39].

### 2.4. Immune System

The Nr4a’s have been extensively studied in the immune system. This subfamily plays an important role in regulating various immune cell types, in particular T cells. It is important to note, again, that many of these studies were completed using the Nr4a1 knock out animal that expresses NTD of Nr4a1, which has been shown to stabilize HIF1α [52]. Some of the observed phenotypes of this mouse, particularly in the immune system, have not been found in the floxed Nr4a1 mice, that result in complete gene deletion. This mouse was often used in studies of acute myeloid leukemia (AML)

#### 2.4.1. Pro-Proliferation

The development of a Nr4a1 floxed, Nr4a2 floxed and Nr4a3 deficient mouse as a triple knockout of the Nr4a’s showed their essential role in immune system regulation. These mice die within 21 days of birth with systemic autoimmunity, decreased Treg populations and decreased peripheral T cell proliferation [55]. They have also been found relevant individually in various immune cells types. In cultured neutrophils, Nr4a2 knock down decreases cell viability [56]. Nr4a3 knock down in mouse dendritic cells presents with decreased functionality, including decreased stimulation of T cell proliferation, which is a key step in the adaptive immune response [57]. 

#### 2.4.2. Anti-Proliferation

Nr4a1 appears to be a negative regulator of T cell proliferation. CD8+ T cell proliferation was inhibited by Nr4a1, through binding to the Irf4 promoter and inhibiting transcription and ultimately Irf4′s proliferative effects in T cells [40]. Nr4a1 knock down also increased dendritic cell function and T cell proliferation to the NF-κB-dependent inflammatory response [41]. While Nr4a3 promotes T cell proliferation through dendritic cells, mice with bone marrow transplanted from Nr4a3 deficient mice have more granulocyte-macrophage precursors and macrophage and dendritic cell progenitors, which is linked to increased plaque formation in atherosclerosis. Nr4a3 deficiency increased RUNX1 expression, a key transcription factor that promotes myeloid progenitor proliferation. Nr4a3 overexpression in turn decreased RUNX1 expression [42].

### 2.5. Cancer

Just as the Nr4a family members has different functions in different tissues, they behave differently in cancers of various origins. For some tumors the Nr4a’s promote proliferation and in others they inhibit it, making modulation of these transcription factors a possible therapeutic target. Angiogenesis is also linked to the Nr4a subfamily, making it another target to limit tumor growth.

#### 2.5.1. Promotes Proliferation

Nr4a1 and Nr4a2 promote lung cancer growth. H460 and Calu-6 cells overexpressing Nr4a1 had greater BrdU incorporation and Nr4a1 knockdown inhibited H460 cell growth [58]. SPC-A1 and H1299 cells had decreased proliferation when Nr4a1 was knocked down [59]. Nr4a2 knockdown in H157 cells blocked drug induced proliferation, while increased Nr4a2 expression correlated with increased Cyclin D1 expression [43]. Interestingly, Nr4a3 knockdown increased BrdU incorporation, tumor weight and decreased TUNEL positive cells in BRE-AS1 expressing H1299 cells [60]. This data suggests that while Nr4a1 and Nr4a2 increase lung cancer proliferation, Nr4a3 has an inhibitory effect. 

Nr4a1 has also been specifically identified to induce proliferation in colorectal cancer, renal cancer, medulloblastomas and thyroid cancer. Two groups showed that Nr4a1 knock down in HCT116 colorectal cancer cells decreased growth and viability [61,62]. Nr4a1 knock down decreased SW620 colony formation, while Nr4a1 overexpression had the opposite effect [63]. Treatment of RKO cells with the Nr4a1 agonist DIM-C-pPhOCH3 lead to decreased proliferation, in contrast with finding with other cell lines [64]. ACHN and 786-O renal cell lines showed that the Nr4a1 antagonists DIM-C-pPhOH and DIM-C-pPhCO_2_Me decreased cell numbers in a dose dependent manner and decreased ACHN xenograft size, suggesting that Nr4a1 also promotes renal cancer proliferation [65]. Nr4a1 overexpression increased medulloblastoma cell growth and viability, while Nr4a1 knock down decreased proliferation [66]. TT cells, a medullary thyroid carcinoma line, had decreased proliferation when Nr4a1 was knocked down [67]. 

Just as in the pancreatic β-cells, the Nr4a’s promote proliferation in pancreatic cancer lines. Nr4a1 knockdown in Panc1 cells had decreased survival and Panc1, MiaPaCa-2 and L3.6pl cell lines treated with the Nr4a1 antagonist DIM-C-pPhOH presented with decreased proliferation [68]. Nr4a2 knock down in BxPC-3 pancreatic cancer cells had decreased cell growth and colony formation [69]. Nr4a2 overexpression increase HTB-52 hepatocellular carcinoma proliferation [70]. Nr4a1 knock down in HeLa cells causes decreased viability and increased growth with Nr4a3 overexpression [71,72]. Nr4a1 is regulated by Pin1 in HeLa cells to express Cyclin D2 and E2F1 and drive proliferation [44]. MCF10A salivary gland cancer cells and mouse submandibular gland cells overexpressing Nr4a3 also showed increased proliferation [73]. Taken together, these data clearly demonstrate a subset of cancers in which Nr4a’s induce proliferation. While this data shows how the Nr4a’s are important for the growth of many cancers, there are many where the reverse is true.

#### 2.5.2. Inhibits Proliferation

The roles of Nr4a1 and Nr4a3 have been studied extensively in AML, where they have similar function. Nr4a1 and Nr4a3 deficient mice develop AML with extensive myeloid cell proliferation due to decreased c-Jun and JunB expression [18]. Nr4a1 floxed and Nr4a3 deficient mice created a less severe AML phenotype but still showed increased hematopoietic stem cell proliferation and decreased bone marrow cellularity. In this state CEBPα, an AML tumor suppressor, was down regulated and Myc genes, STAT1, inflammatory proliferation genes like IL-6 and phosphorylation of PKB/AKT and ERK1/2 all increased [46]. Another study used both Nr4a1^-/-^ Nr4a3^+/-^ and Nr4a1^+/-^ Nr4a3^-/-^ mice, which increased neutrophil and monocyte proliferation, as well as an increased the BrdU incorporation level in hematopoietic stem cells, multipotent progenitor cells and myeloid progenitors [74]. These studies show that loss of Nr4a1 and Nr4a3 results in impaired immune regulation as well as AML progression. 

Studies have further examined the roles of Nr4a1 and Nr4a3 in AML cell lines and other immune cell cancers. Nr4a1 downregulates proliferation associated genes such as c-MYC, BCL2, CBFA2T3 and CSF1R and the myeloid transcription factor PU.1 in Kasumi-1 cells [47]. Nr4a1 and Nr4a3 overexpression decreased Kasumi-1 proliferation and cell viability. Analysis showed that Myc and Bcl2 were down regulated by Nr4a1 and Nr4a3 while TGF-B was upregulated, increasing p57 levels [48]. The pharmacological compounds DHE and ALP are sufficient to block AML tumor growth by inducing Nr4a expression and down regulating c-Myc [75] Nr4a3 overexpression decreased SuDHL4 B cell line proliferation and xenograph growth. Validating this, Nr4a3 knock down increased cell viability of the U2932 and SuDHL4 cell lines [76]. The observed AML phenotypes in multiple Nr4a knockout mice draws a strong tie to their role in the immune system these genes play and, in a manner, that none of the other cancer tissues can quite match, making this a strong area for continued research. 

In addition to their role of inhibiting proliferation in hematopoietic based malignancies Nr4a1 overexpression in UM-UC-3 bladder cancer cells decreased androgen stimulated proliferation, while Nr4a1 knockdown increased growth [77]. Similarly, Nr4a1 overexpression in Ishikawa cells, an endometrial cancer line, decreased proliferation, while Nr4a1 knock down increased proliferation [78]. These data clearly demonstrate a subpopulation of tumors where Nr4a expression inhibits proliferation. 

#### 2.5.3. Varied Results

While the Nr4a subfamily may be clear targets in the malignancies discussed this far, there are a few cancers where their role is conflicting, making them less ideal therapeutic targets. The effects of the Nr4a subfamily on breast cancer proliferation is not consistent. MCF-7, MDA-MB-231 and SKBR3 cell lines had decreased cell number and tumor weigh when treated with the Nr4a1 antagonists DIM-C-pPhCO2Me and DIM-C-pPhCN, as well as with Nr4a1 knock down [79]. MDA-MB-231 cells also have decreased proliferation with Nr4a1 overexpression due to JNK1, c-Jun and Cyclin D1 downregulation [45]. MCF-10A cells had no change in proliferation when overexpressing Nr4a1 and measurements across many breast cancer tumors found Nr4a1 expression to be inconsistent, suggesting that Nr4a1′s role in breast cancer may be tumor specific [80]. MDA-MB-468 and MDA-MB-231 lines also had lower xenograft weights with Nr4a2 knockdown [81]. 

Nr4a’s role in prostate cancer is also conflicting. Nr4a1 knock down decreased PC3 cell line proliferation [82], DU145 and PC3 cells were also Nr4a3 sensitive, with Nr4a3 overexpression decreasing proliferation and tumor size and Nr4a3 proliferation increasing proliferation [83]. These data suggest that Nr4a1 is pro-proliferative while Nr4a3 is anti-proliferative. This may be similar to primary tissue where Nr4a1 and Nr4a3 play opposing roles to regulate proliferation.

#### 2.5.4. Angiogenesis

While the Nr4a’s role in tumor proliferation is being studied as a therapeutic target it is also being examined for their role in angiogenesis and endothelial cell proliferation. VEGF-A induces Nr4a1 expression and Nr4a1 overexpression in the absence of VEGF-A is sufficient to induce primary umbilical vein endothelial cell proliferation to the same level of VEGF-A alone. Furthermore, Nr4a1 mutants blocked the VEGF-A mediated proliferation effect. Nr4a1 upregulates Cyclin A, Cyclin D1, PCNA and E2F in vasculature endothelial cells [49]. Histamine and serotonin are both sufficient to induce endothelial cell proliferation, however cells deficient in Nr4a1 are resistant to these treatments [84]. Nr4a1 knockdown decreased VEGF induced DNA synthesis in human umbilical vein endothelial cells and when Nr4a1 was knocked down in the presence of VEGF there were fewer vessels per square millimeter in microscopic analysis, suggesting that VEGF acts through Nr4a1 in angiogenesis [85]. As angiogenesis is vital for a tumor expansion and metastasis, it be a potential target to limit the tumor growth regardless of tissue of origin.

### 2.6. Other Tissues of Note

#### 2.6.1. Pro-Proliferation

Among all of the tissues included there are a few more to be noted where the Nr4a’s have an effect. In an intestinal ischemia mouse model, the mice treated with the Nr4a2 activator C-DIM12 presented increased Ki67+ cells and better recovery [86]. Nr4a1 knock down in IEC-6 intestinal epithelial cells had decreased proliferation, while overexpression increased proliferation by inhibiting p21^CIP1^ expression [50]. Nr4a1 also promotes spermatogonium development in human primary Sertoli cells by inducing expression of GDNF [87]. Synoviocyte proliferation is part of the pathogenesis of arthritis. Nr4a2 overexpression increased K4IM synoviocyte proliferation, while Nr4a2 knock down decreased proliferation, indicating Nr4a2 as a possible arthritis regulator [88].

#### 2.6.2. Anti-Proliferation

Knockdown of Nr4a1 and Nr4a2 by in mesenchymal stromal cells increased cell cycle progression and overexpression impeded proliferation by inducing a G1 cell cycle block [89]. Nr4a1 knock down increased NIH3T3 fibroblast cell growth [90]. Overexpression of each of the Nr4a genes decreased leiomyoma smooth muscle cells, part of uterine fibroids, proliferation rate by decreasing TGFβ3, SMAD3 and collagen genes 1A1, 6A1, 6A2 and 16A1 expression [51].

#### 2.6.3. Varied Results

Neural tissue presented mixed results with regards to Nr4a2 and proliferation. Nr4a2 overexpression in primary neural stem cells from the olfactory bulb led to fewer BrdU^+^ cells but when rat adult hippocampal neural precursors were treated with the Nr4a2 agonist amodiaquine, BrdU incorporation and viability increased [91,92].

These data clearly demonstrate that the Nr4a’s play a critical role in regulating cellular proliferation. The Nr4a’s clearly have a tissue specific effect, where they may induce proliferation in one tissue while inhibiting in another. This suggests differences in chromatin accessibility, either due to changes in binding partner expression, post translational modification or chromatin modifications. These data support the ongoing studies to understand mechanisms by which the Nr4a’s induce or inhibit proliferation as potential therapeutic intervention for various conditions.

## 3. Apoptosis

The second major function of the Nr4a nuclear hormone receptors is regulating cellular apoptosis. The role of the Nr4a’s in controlling apoptosis is complex and present across several tissues. This section will focus on the role of the Nr4a’s on apoptosis, focusing on transcriptional changes in the intrinsic and extrinsic apoptosis pathways, as well as non-genomic functions of the Nr4a nuclear receptors that impinge on apoptosis. The genes that are transcriptionally regulated by the Nr4a’s that are essential for cellular apoptosis are summarized in Table 2. 

### 3.1. Intrinsic Apoptotic Pathway

There are two main methods that regulate apoptosis in mammalian cells. They are the intrinsic pathway or mitochondrial signaled apoptosis pathway and the extrinsic pathway or death-receptor mediated pathway. Both involve amplification of proteolytic cascades which leads to apoptosis [104,105]. The intrinsic pathway is activated by cellular stressors that increase mitochondrial permeability. These stressors are recognized by several internal proteins that signal to the mitochondria. The Bcl-2 family is most commonly used in this interaction. The ratio of pro-apoptotic and anti-apoptotic Bcl-2 proteins regulate the pathway. When pro-apoptotic Bcl-2 proteins accumulate, they translocate to the mitochondria and form pores in the mitochondrial membrane. This causes cytochrome c release, caspase-9 activation and eventual cell death [106,107,108]. Understanding how the Nr4a receptor family regulate these pathways will be addressed. 

#### 3.1.1. Liver

Liver fibrosis is a wound healing response caused by over accumulation of extracellular matrix proteins. This leads to decreased liver function and eventually cirrhosis [109]. Nr4a2 overexpression in HSC decreases liver fibrosis [29]. Nr4a2 regulates ERK1/2 and p38 expression in HSC leading to apoptosis. Rats overexpressing Nr4a2 treated with dimethylnitrosamine, which induces liver fibrosis, are protected from liver damage. Nr4a2 overexpression induces apoptosis in the cirrhotic tissue [29]. This shows that Nr4a2 protects healthy liver tissue from becoming fibrotic while also inducing apoptosis in fibrotic tissue via the ERK1/2 and p38 pathway.

#### 3.1.2. β-Cell

The occurrence of type 2 diabetes is closely linked to the loss of pancreatic β-cells and their function [22,110]. Pancreatic β-cells are sensitive to hyperglycemia and hyperlipidemia, which alter Ca^2+^ concentrations in pancreatic islets, leading to increased endoplasmic reticulum (ER) stress and eventually apoptosis via the intrinsic apoptosis pathway [110,111]. Thapsigargin (TG) and palmitic acid (PA) are known inducers of β-cell ER stress. MIN6 cells and primary C57BL/6J mouse islets treatment with TG and PA caused increases in Nr4a1 and C/EBP homologous protein (CHOP) levels in a time-dependent manner [95]. Nr4a1 overexpression in MIN6 cells and islets increased cell survival rates as measured by MTT, TUNEL assay and Caspase3 cleavage. Survivin protein binds to pro-caspase 3 and prevent Caspase 3 activation, reducing cell death [112]. Nr4a1 directly regulates Survivin expression through promoter binding. Nr4a1 knockdown in MIN6 cells treated with TG and PA caused decreased cellular viability via decreased Survivin expression, leading to increased Caspase3 activation and increased CHOP protein. This suggests that Nr4a1 protects the β-cell and primary islets against ER stress by directly upregulating Survivin and indirectly downregulating CHOP.

Proper β-cell function is central to the pathogenesis of type-2 diabetes mellitus [113,114,115]. Oxidative stress is commonly found in the β-cells of diabetic patients and prevention of this stress is protective in pancreatic β-cells [116]. MIN6 cells treated with H_2_O_2_ increases Nr4a1 levels and increased Caspase3 cleavage [96]. Nr4a1 overexpression in MIN6 and primary mouse islets cells protects from reactive oxygen species (ROS) damage and increases cellular viability. Expression experiments determined that Nr4a1 overexpression upregulated anti-apoptotic genes (WT1 and BCL-2) and ROS scavenger genes (SOD1). Genetic analysis showed a NBRE binding sequence in the WT1 promoter, ChIP showed that Nr4a1 directly regulates WT1 expression and WT1 knockdown decreased BCL-2 and SOD1 expression. Therefore, Nr4a1 protects primary islets from ROS by directly altering WT1 expression and indirectly increasing BCL-2 and SOD1 expression.

Nr4a3 expression has recently been shown to correlate with cytokine mediated β-cell death [10]. Nr4a3 was upregulated in Ins-1 cells and human islets cultured with IL-1 β and TNFα. This resulted in increased apoptosis as measured by TUNEL staining, DNA fragmentation and cytochrome c release. Knock down of Nr4a3 impaired cytokine mediated apoptosis and re-expression of Nr4a3 induced apoptosis. Together these data suggest that Nr4a1 is anti-apoptotic and Nr4a3 is pro-apoptotic in the pancreatic β-cells. 

#### 3.1.3. Cancer

As previously stated, cytochrome c contributes to apoptosis by initiating the caspase cascade upon its release into the cytosol [117,118,119]. Nr4a1 knockdown significantly decreased apoptosis in prostate cancer cells. A GFP-Nr4a1 fusion protein was used to track location of Nr4a1 during apoptotic treatment. Nr4a1 translocates from the nucleus to the mitochondria when treated with several apoptotic drugs [120]. Prostate cancer cells treated with leptomycin B, which blocks nuclear export [121] and apoptotic drugs, showed decreased cytochrome c release due to decreased Nr4a1 mitochondrial binding [120]. Interestingly, deletion of the DNA binding domain (DBD) of Nr4a1 had no effect on cytochrome c release. It was determined that a 152-amino acid residue from the NH_2_-terminus and 96 amino acids from the COOH-terminus are critical for Nr4a1 mitochondrial targeting. When Nr4a1 was altered to target the plasma membrane [122] or the ER [123] apoptosis was decreased. Similar experiments were done in rats exposed to ischemia in vivo and in primary melanoma tissue [124,125]. Nr4a1 levels were decreased in primary melanoma tissue, possibly leading to protection from apoptotic treatments. Nr4a1 co-localizes with Bcl-2 in the mitochondria in prostate cancer cells, melanoma cells and gastric cancer cells [125,126,127,128] These data confirm that Nr4a1 has extra-nuclear functions, independent of DNA binding, as part of the intrinsic apoptotic pathway to induce cytochrome c release. Furthermore, several studies show that the Nr4a family have significant non-genomic function in the body as described [52,93,100,101,102,103,129]. Taken together, this shows that while the Nr4a’s primarily function as genetic regulators they have non-genomic functions that are integral for cellular apoptosis and function. The use of the Nr4a1 floxed mouse and the Nr4a1 full body NTD-Nr4a1 mouse could be leveraged to better determine the non-genomic roles of Nr4a1 [101]. 

Post-translational modifications (PTMs) alter protein function [130,131]. Phosphorylation of Nr4a1 at Ser351 by Akt blocks mitochondrial targeting in H460 lung cancer cells and BGC-823 gastric cancer cells [127,132,133]. Interestingly, in BGC-823 cells Ser351 phosphorylation blocks both nuclear export of Nr4a1 and mitochondrial targeting of cytosolic Nr4a1. The mitochondrial interaction of Nr4a1 in these cancer cells was independent of its DBD, supporting previous data [120]. However, in BGC-823 cells Akt phosphorylation only occurred on the N-terminus of Nr4a1 [127]. Nuclear phosphorylation of Nr4a1 at Ser105 induces export and mitochondrial targeting [133,134]. Akt phosphorylation of Nr4a1 decreases apoptosis and cytochrome c release. Together, these data show that Nr4a1 is regulated by PTMs and that it is integral for apoptosis in cancer cells.

Acute myeloid leukemia is caused by an increase in immature myeloid progenitors and the emergence of leukemia initiating cells. Properties and structure of these cells makes AML difficult to treat. The Nr4a family control regulation of hematopoietic stem cell development [18]. Nr4a1 and Nr4a3 acute expression is sufficient to decrease cellular viability in Kasumi-1 cell lines in a DNA binding domain dependent manner [48]. Nr4a1 and Nr4a3 have a strong inverse correlation to several MYC target genes in Kasumi-1 cells, including Bcl-2 and TNF [48]. Nr4a1 and Nr4a3 also regulate several different types of lymphoma, including non-Hodgkin’s and B-cell. Human samples from cancer patients were analyzed for the Nr4a family and their downstream targets [135,136,137]. They found that cytoplasmic localization of Nr4a1 increased overall life expectancy of B-cell lymphoma patients through decreased caspase 3 cleavage and ERK1/2 levels [136]. Furthermore, both Nr4a1 and Nr4a3 levels were decreased in Non-Hodgkin’s lymphoma, as well as FasL and TRAIL proteins, which could result in decreased intrinsic and extrinsic apoptotic signaling. Decreased Nr4a1 showed a significant association with poor cancer-specific survival, suggesting that Nr4a1 could be used as an independent prognostic indicator [135]. In SuDHL4 lymphoma cell lines, Nr4a1 overexpression led to increased cleaved caspase 3/7 levels and Annexin V showing an increase an apoptosis. This increase in apoptosis was caused by increased pro-apoptotic proteins, TRAIL, Bim and Puma, suggesting that Nr4a1 acts as an activator on genes responsible for apoptosis in lymphoma cells [135]. Nr4a3 was also found to bind to p53 and induce apoptosis in breast and lung cancer cells and that high levels of Nr4a3 correlated to better survival in these patients [137]. This shows that Nr4a1 and Nr4a3 controls induction of apoptosis in different lymphoma cells.

### 3.2. Extrinsic Apoptotic Pathway

The extrinsic pathway begins with attachment of ligands to the extracellular domain of transmembrane proteins [138]. Ligand binding signals the intracellular death domain to bind with its corresponding protein motif. These adaptor proteins have a death effector domain which interacts with the death effector domain of procaspase-8. This interaction activates a death inducing signaling complex which catalyzes procaspase-8. Cleaved caspase-8 activates effector caspases which cause cell death by damaging the nucleus and other internal organelle [139,140,141].

#### 3.2.1. Muscle

The inhibitor of apoptosis (IAP) family of proteins are critical in the regulation of apoptosis [142,143]. IAPs promote pro-survival signaling pathways and prevent apoptosis by interfering with caspase activity [143]. Nr4a3 overexpression protects vascular smooth muscle cells (VSMC) from apoptotic treatment. Hypoxia and the hypoxia mimetic cobalt chloride increased Nr4a3 levels in VSMC. This regulation was determined to be dose and time dependent. Nr4a3 directly regulates cIAP2 expression in VSMC through an NBRE binding site [94]. Knockdown of cIAP2 removes Nr4a3 driven protection. This shows that Nr4a3 protects VSMC from caspase activity by increasing expression of cIAP2.

#### 3.2.2. Immune System 

The Nr4a family also induces apoptosis in certain cell types. This has been well documented in T-cells. Induced apoptosis in A04H5.3 T-cells causes an increase in Nr4a1 RNA and protein expression and Nr4a1 dominant negative mutant was protected from cell death [144]. This was also confirmed in vivo using C57Bl/6 transgenic mice which constitutively expressed wildtype Nr4a1 or the dominant negative mutant. They found that Nr4a1 dominant negative mice had lower levels of thymocytes, mature T cells and decreased tolerance while the constitutively expressed wildtype mice were unable to perform antigen-induced negative selection of the T cells [145,146]. While the direct Nr4a1 target genes are still unknown in T-cells there is a correlation between FasL and Nr4a1 expression [97,147]. This shows a complex regulatory network in the Nr4a family that has not been fully determined.

Macrophage treated with LPS and simvastatin undergo a caspase independent apoptotic pathway [148]. Treating RAW 264.7 cells with farnesyl transferase inhibitors decreased LPS and simvastatin mediated apoptosis, suggestion that farnesyl proteins are responsible for apoptosis [149]. Simvastatin treatment alone gradually increases Nr4a1 mRNA levels overtime. RAW 264.7 cells treated with a dominant negative Nr4a1 showed significantly decreased DNA fragmentation and decreased disruption of mitochondrial membrane potential. This is caused by decreased levels of BAX translocation to the mitochondria in cells treated with farnesyl inhibitors [150]. This data shows that Nr4a1 is crucial for non-caspase driven apoptosis pathway and that N4a1 plays a role in mitochondrial membrane potential in RAW 264.7 cells.

T-cells expressing chimeric antigen receptors (CAR) targeting human CD19 have exhibited impressive clinical efficacy against B cell cancers [151]. CAR T-cell have been less effective against solid tumors due to an increase in antigen stimulation and decrease effector function [152]. CAR T-cells with deficiencies in Nr4a1, Nr4a2 and Nr4a3 showed improved tumor cell death and increased effector function [153]. This improvement is caused by downregulation of CD8^+^ T-cell inhibitory receptors PD-1, TIM3 and CTLA4. This function is important for healthy T-cell formation but it is inhibitory for CAR T-cells and leads to dysfunction and exhaustion. This shows that the Nr4a family plays in important role for CAR T-cell function against solid tumors.

#### 3.2.3. Other

The effect of the Nr4a family over the extrinsic apoptosis pathway is very complex and varies depending on cell type and stimulus. H_2_O_2_ treatment is commonly used to measure cellular viability as ROS is sufficient to induce apoptosis [98,154,155]. Treatment of human umbilical cord fibroblast (HUC-F2) with H_2_O_2_ for 4 h increased Nr4a1 mRNA levels. Knockdown of Nr4a1 decreased cellular viability by increased activated caspase 3 and 8 levels [99]. Therefore, in HUC-F2 cells Nr4a1 functions in a protective, anti-apoptotic role. Similar data have been seen in several different human cancer cell lines, including Panc-1, L3.6pl, MCF7 and RKO, using both siRNA and 1,1-bis(3′-indolyl)-1-(*p*-anisyl) methane treatments [68,156,157]. Nr4a1 directly regulates TXNDC5 expression which protects Panc-1 cells from ER-stress mediated apoptosis [157]. In primary colon tumor tissue, Nr4a1 expression levels are elevated [68]. Although, Nr4a1 directly regulates tumor necrosis factor–related apoptosis-inducing ligand (TRAIL) protein and PDCD1 in RKO cells. Nr4a1 knockdown in C57BL/6 nude mice with RKO cells xenografts had decreased tumor volume and final tumor weight [64]. This shows that Nr4a1 plays an important role across different tissues by protecting cells from apoptosis.

These data clearly indicate that the Nr4as play a critical role in regulating apoptosis across several different tissues. There does not appear to be a tissue specific effect of these proteins or consensus across the protein family in response to similar treatment. What we can conclude is that their regulatory network is complicated and more research needs to be done fully illuminate their function in regulating apoptotic pathways and future use for therapeutic treatments.

## 4. Fuel Utilization

A third major function of the Nr4a nuclear hormone receptors is controlling expression of genes associated with fuel utilization. The function of the Nr4a’s in controlling cellular fuel utilization is a function conserved from *C. elegans* to man, where studies have shown its function in glucose metabolism, lipid metabolism, TCA cycle and oxidative phosphorylation. This section reviews the role of the Nr4a’s in fuel utilization, focusing on effects in glucose metabolism, lipid metabolism and mitochondrial function. The genes regulated by the Nr4a’s that are essential for fuel utilization are summarized in Table 3.

### 4.1. Glucose Metabolism

The *C. elegans* Nr4a ortholog is NHR-6. NHR-6 has preferential binding to promoters associated with glucose metabolism [185]. Similar results have shown that Drosophila Nr4a (DHR38) is essential for carbohydrate metabolism, glycogen storage and ethanol metabolism [186,187]. Finally, animal models of insulin resistance and mismanaged glucose metabolism, such as ob/ob and db/db mice or STZ treated or ZDF rats, demonstrate a significant decrease in Nr4a expression in muscle and adipose tissue [174]. These data demonstrate the intimate link between Nr4a transcriptional activity and glucose metabolism. In this section we will review the role of the Nr4a’s in glucose metabolism across various tissues.

#### 4.1.1. Liver

Given the connection between Nr4a’s and glucose metabolism, the liver is an organ of immense interest. Full body Nr4a1 knock out mice present contradictory findings when comparing liver insulin sensitivity. Nr4a1 knock out mice fed a control diet had improved insulin sensitivity, while mice fed a high fat diet presented impaired insulin sensitivity, as demonstrated by hepatic glucose production rate and glucose infusion rate during euglycemic clamp studies [20]. Expression of the glycolytic genes Bpgm and Pgk1 was upregulated in the liver from high fat fed Nr4a1 knock out mice and expression of G6pc was downregulated, while expression of the gluconeogenic genes Fbp1 and Pgc1a was upregulated. Interestingly, expression of the G6Pase, which allows glucose to be released from hepatocytes, was downregulated in Nr4a1 knock out hepatocytes under both feeding regimes [20]. It is important to note, however, that a recent study using Cre/flox mediated Nr4a1 deletion demonstrated that the previously published full body Nr4a1 knock out produces a N-terminal domain only truncated Nr4a1 that is sufficient to interact with and stabilize HIF1α [52]. This report demonstrates that some of the phenotypes previously thought to be due to the genomic effects of Nr4a1 may in fact be due to non-genomic functions. Given this result, it is important to validate many of these findings using this model.

Primary hepatocytes treated with glucagon increase expression of Nr4a1, Nr4a2 and Nr4a3, in a cAMP dependent process [158]. Nr4a1 hepatic overexpression results in increased expression of Fbp1, Eno3, G6pc, Fbp2 and Glut 2 [158]. Similarly, Nr4a2 and Nr4a3 overexpression also induce expression of these target genes, resulting in increased gluconeogenesis. The Nr4a mediated induction of these genes correlates with direct binding of Nr4a1, Nr4a2 or Nr4a3 to NBRE sites in the target gene promoters [158].

A recent study demonstrated a direct connection between Nr4a1 and Gyk [188]. Gyk was shown to inhibit expression of two Nr4a1 liver target genes, Eno3 and ApoA5, demonstrating that increased levels of Gyk decreased expression of these targets through sequestering Nr4a1. This resulted in decreased Eno3, G6Pase and Fbp1 expression and ultimately decreased circulating blood glucose levels [188]. These results were validated with Gyk overexpression in the STZ treated mice and in db/db mice. HepG2 Nr4a1 overexpression was sufficient to decrease expression of the gluconeogenic enzyme G6pc and PEPCK, while Nr4a1 knock down increased expression of these same target genes [161]. Treatment of mice with the conjugated linoleic acid (CLA) trans-10,cis-12-CLA resulted in increased expression of Nr4a1, Nr4a2 and Nr4a3, as well as increased expression of the gluconeogenic genes G6pc, Eno3, PEPCK and PC [159]. Finally, treating mice with the Nr4a1 agonist Csn-B is sufficient to increase circulating blood glucose levels and induce expression of G6pc and Fbp1 [189]. Together these data demonstrate that Nr4a’s (particularly Nr4a1) plays a critical role in regulating expression of glycolysis and gluconeogenesis in hepatocytes.

#### 4.1.2. Muscle

The Nr4a family directly effects muscle glucose utilization pathways. Chao et al. demonstrated that Nr4a1 is expressed in muscle in response to β-adrenergic signaling and that blocking this signal was sufficient to decrease expression of genes associated with glucose uptake (Glut4), glycolysis (Pfkm) and glycogenolysis (Pygm) [163]. Furthermore, Nr4a1 overexpression in C2C12 muscle cells or in electroporated primary muscle cells was sufficient to induce expression of these same gene classes. Finally, Nr4a1 knock out muscle demonstrated downregulation of these genes and luciferase and EMSA demonstrated Nr4a1 binding to the promoter of Glut4 and Eno3 [20]. Subsequent studies from the same group demonstrated that full body Nr4a1 knock out mice fed a high fat diet have impaired glucose tolerance and elevated circulating insulin levels [20]. Their data demonstrated muscular insulin resistance, decreased Glut4 expression, decreased insulin receptor phosphorylation and decreased expression of glycolytic genes such as Eno3, Bpgm and Pgk1. Further findings demonstrated that loss of Nr4a1 in muscle significantly impairs myofiber size and regeneration [166,167]. Nr4a1 overexpression or induction with metformin or the Nr4a1 pharmaceutical ligand DIM-C-pPhOH and its derivatives in C2C12 muscle cells increased expression of metabolic genes associated with glucose uptake, glycolysis and glycogenolysis, such as Glut4, PHKG1, PGAM2, Eno3, Aldo1 and Pygm [172]. This study demonstrated enhanced glucose uptake and showed that knock down of Nr4a1 impaired the induction observed with either metformin or DIM-C-pPhOH treatment [172]. Similarly, neonatal rat ventricular myocytes overexpressing Nr4a1 presented with elevated glycolysis, as well as induction of the hexosamine pathway branch through GFPT2 induction, resulting in enhanced O-GlcNAcylation of STIM1, ultimately affecting cardiac performance [171].

Nr4a3 results in similar changes in glucose metabolism gene expression. Muscle specific Nr4a3 overexpression mice fed a control diet presented with decreased epididymal, inguinal, brown and total adipose mass [169]. This corresponded with significantly lower circulating leptin levels. When these mice were fed a high fat diet, the animals had decreased weight gain, adipose mass and circulating blood glucose [169]. Significant upregulation of glycolytic genes in the Nr4a3 overexpressing muscle was observed, including induction of HK1, Pfk-1, GAPDH, Pgam2, Eno3 and Pkm [169]. In addition, components of the malate aspartate shuttle, including MDH and AST, were upregulated, as was Pdh, with downregulation of the glycerol phosphate shuttle and decreased NAD+/NADH ratio, indicative of more efficient ATP production [169]. Nr4a3 overexpression in muscle also resulted in increased expression of the glycogenic genes Glut4, HK2, Stbd1 and Gys1, as well as downregulation of the glycogenolytic gene PPP1R1A [169]. This corresponded with increased muscular glycogen content [170]. Together, these data clearly demonstrate that Nr4a1 and Nr4a3 positively regulate glycolysis and glycogenesis within muscle cells.

#### 4.1.3. Adipose Tissue

Nr4a’s also play an important role in adipose tissue glucose utilization. The insulin sensitizing drugs pioglitazone and troglitazone increase adipose tissue glucose uptake and increase expression of Nr4a1 and Nr4a3 [174]. Similarly, insulin induces Nr4a expression in the 3T3-L1 adipocytes. Finally, overexpression or knock down of Nr4a3 is sufficient to impair adipocyte glucose uptake and overexpression of Nr4a3 causes increased Glut4 expression and transport to the plasma membrane [174]. Interestingly, human adipose samples from obese subjects prior to bariatric surgery demonstrate significantly elevated expression of Nr4a1, Nr4a2 and Nr4a3 and the expression level drops one year after surgery to levels observed in lean subjects [175]. These data support the hypothesis that Nr4a’s control adipocyte glucose metabolism.

#### 4.1.4. β-cells

Loss of Nr4a1 or Nr4a3 but not Nr4a2, in the 832/13 INS-1 β-cell line decreased expression of the glycolytic gene Eno1 [177]. No changes were observed, however, with Aldoa or Pgk1. Similar results are found in the MIN-6 cell line, where Nr4a1 expression was induced by culturing with palmitate, which increased Eno3 expression [179]. Nr4a1 is induced in β-cells in response to palmitate or oleate exposure [190]. Culturing INS-1 β-cells in the presence of oleate, followed by mass spectrometry to define changes to the β-cell proteome demonstrates downregulation of the glycolytic genes Eno1, Eno3, GAPDH and Pgk1 [178]. These data demonstrate that Nr4a1 and Nr4a3 are sufficient to regulate glycolytic gene expression in the pancreatic β-cell, which impinges on glucose utilization and insulin secretion.

#### 4.1.5. Immune System

Changes in fuel metabolism are essential for immune cell differentiation and clonal expansion. A recent study of T-cell activation demonstrated that Nr4a1 knock out T-cells have greater rates of cellular proliferation [180]. This corresponds with increased basal respiration, maximal respiration, glycolysis and glycolytic capacity. They demonstrated upregulation of genes involved in glycolysis, including Hk2, Aldoa and Aldoc [180]. In addition to inducing glycolytic changes, changes were observed in glycogenesis (Gbe1) and glycogenolysis (Agl) [180]. A previous study demonstrated that loss of Nr4a1 impairs Treg differentiation, which corresponds with decreased glycolytic gene expression [191]. Interestingly, loss of Nr4a1 expression in macrophages impairs glycolysis in the LPS stimulated state [181]. This corresponds with decreased lactate production from anaerobic glycolysis. This shift to glycolytic metabolism during macrophage activation is essential for proper production of proinflammatory cytokines and nitric oxide, which is deregulated in Nr4a1 KO mice, resulting in elevated atherosclerotic plaques. Finally, a floxed Nr4a1 mouse model demonstrated decreased expression of Glut1 and Pdk1 in bone marrow [52]. This model which gives complete Nr4a1 deletion, rather than production of the NTD domain only Nr4a1 emphasizes that Nr4a1 (rather than HIF1α or another Nr4a1-NTD binding partner) regulates expression of glycolytic genes. Currently, however, these data suggest that Nr4a1 impedes glycolytic respiration in T-cells, a critical change during T-cell activation.

#### 4.1.6. Cancer

Similar results are described in acute myeloid leukemia (AML) cells from patients and using the NB4 and THP1 cell lines. AML cells from patients have significantly decreased Nr4a1 expression as compared to healthy donors. siRNA mediated knockdown of Nr4a1 in the NB4 cell line caused increased proliferation, Glut1 and Ldha expression and lactate levels. These data suggest that in AML cells, loss of Nr4a1 results in enhanced glucose uptake and anaerobic glycolysis [182]. Treatment with dihydroergotamine (DHE) is sufficient to induce Nr4a1, Nr4a2 and Nr4a3 expression in AML cell lines. DHE treatment also results in induction of Eno3 expression [75]. Furthermore, ChIP-seq of Nr41 binding in AML cells demonstrates strong binding to promoters of various glycolytic genes, including Eno3 [47]. These data clearly demonstrate Nr4a1 regulating glycolytic gene expression in AML cells.

### 4.2. Lipid Metabolism

Where glucose metabolism focuses on use and production of fuel in the fed state or short-term fuel storage in the form of glycogen, lipid metabolism focuses on the production and usage of the long-term fuel storage form. The Nr4a’s play a role in liver, muscle and adipose tissue controlling the production and usage of lipids.

#### 4.2.1. Liver

Nr4a1 full body knock outs have increased liver triglycerides, cholesterol and hepatic steatosis in response to high fat feeding [20]. This corresponds with increased even-chain acyl carnitine species, increased hepatic Lpl expression and decreased hepatic Ehhadh expression [20]. Hepatic adenoviral mediated Nr4a1 overexpression decreased circulating triglyceride levels, increased LDL levels and decreased HDL levels [160]. This correlates with significantly decreased expression of SREBP1c, FAS and Gpam. In addition, significant downregulation of lipid metabolism genes is observed with Nr4a1 overexpression [160]. Finally, Nr4a1 or Nr4a2 overexpression increased CPT1a in HEPG2 cells, permitting greater fatty acid mitochondrial translocation [162]. These data suggest that Nr4a1 and Nr4a2 push hepatocytes to greater fatty acid production and utilization.

#### 4.2.2. Muscle

Nr4a1 knock down in C2C12 muscle cells decreases triglyceride hydrolysis, as measured by glycerol release [164]. This corresponds with decreased expression of the genes Glut4, CD36, AdipoR2, UCP2 and UCP3 [164]. Nr4a3 overexpression in muscle increased beta-oxidation. This corresponds with increased CRAT, ACSL, ACDH, ECH and 3-KCT expression, all needed for beta-oxidation [169,170]. Nr4a3 loss in C2C12 cells impairs palmitate oxidation and increases anaerobic glycolysis [21]. This corresponded with decreased expression of PGC-1α, PGC-1β, Lipin1α, Pdp1c and Pdp1r [21]. Conversely, treatment with the β-adrenergic activator formoterol enhances these genes associated with oxidative metabolism, presumably through Nr4a3 activation [21]. These data demonstrate that Nr4a1 and Nr4a3 are necessary for muscle lipid metabolism.

#### 4.2.3. Adipose Tissue

The role of Nr4a’s in adipocytes presents conflicting results. Loss of Nr4a1 in 3T3-L1 preadipocytes does not impair adipogenesis or lipid accumulation [192]. This is in contrast to a second report demonstrating that isoalantolactone, which inhibits Nr4a1 activity, decreases 3T3-L1 lipid accumulation [193] and subsequent studies demonstrating that Nr4a1 is essential for adipocyte progenitor function [194]. Finally, Nr4a1 induces p53 and GATA2 expression, which inhibits SREBP1c and PPARγ expression and impairs adipogenesis [173]. Therefore, the current data regarding Nr4a’s and adipogenesis is conflicting.

#### 4.2.4. Cancer

While most of the effects of Nr4a1 are directly linked to gene transcription, Nr4a1 does have non-genomic effects that can impinge on lipid metabolism. In melanoma, Nr4a1 translocates to the mitochondria and stabilize TPβ of the mitochondrial trifunctional protein through direct binding. This binding increasing TPβ activity and increase fatty acid oxidation [183]. Similarly, Nr4a2 induction induces fatty acid oxidation in colorectal cancer [184]. It does this by binding to the promoters and inducing expression of Acox, Cpt1m, Fabp2 and Fabp4.

### 4.3. Mitochondrial Function

Various studies have shown the direct link between Nr4a’s and mitochondrial production and fuel oxidation. This encompasses transcriptional regulation of components of β-oxidation, TCA cycle and the ETC. This section focuses on the regulation controlled by the Nr4a’s in various metabolically active tissues.

#### 4.3.1. Liver

Loss of Nr4a1 impedes high fat diet mediated induction of non-alcoholic fatty liver disease [195]. This was shown to be due to a decrease in high fat diet mediated mitochondrial fission, oxidative stress and calcium overload [195]. These findings demonstrated decreased mitochondrial respiration in primary hepatocytes due to exposure to elevated levels of palmitate and through Nr4a1 upregulation.

#### 4.3.2. Muscle

Nr4a1 overexpression in C2C12 cells resulted in increased mitochondria number [167]. The increased expression of TCA and ETC components corresponds with enhanced glucose tolerance. Nr4a1 overexpression in muscle also resulted in increased levels of NADH-TR diaphorase, Cox1 and Sdhb, all of which result in increased mitochondrial activity [165]. In addition, these mice had greater mitochondrial DNA concentrations, suggesting increased mitogenesis. Muscle had increased Pdh phosphorylation, suggesting decreased pyruvate flux (and supported by elevated lactate levels). The muscle presented with increased fatty acid oxidation and a shift from mitochondrial pyruvate metabolism. The mice demonstrated increased muscle glycogen and increased glycogenesis. The Nr4a1 overexpressing animal muscle had greater mitochondrial respiration, increased expression of Complex 1 and ultimately increased muscle strength [165]. Similarly, C2C12 cells and primary muscle with Nr4a1 knock down demonstrated decreased Ucp3 expression [164]. Mice selected for running capacity have elevated Nr4a1 and Nr4a3 expression in soleus and EDL muscle [168]. Muscle samples from these mice had elevated expression of the Nr4a targets CD36 and Ucp3, as well as the ETC components Complex 1, Complex 2, Complex 3, Complex 4 and Complex 5. Nr4a3 overexpression in muscle increases TCA cycle flux. This corresponds with increased expression of Idh3, Ogdh, Scs, Sdhb, Fh and Mdh [169,170]. Increased levels of mitochondrial ETC components are also upregulated in muscle with Nr4a3 overexpression, including Atp5a1, Uqcr2, Cox1, Sdhb and Ndufb8 [170]. Together, the data is very clear that Nr4a1 and Nr4a3 are essential for muscle mitochondrial content and fuel oxidation.

#### 4.3.3. Adipose Tissue

Using HIB-1B brown adipocytes and brown adipose tissue from warmth and cold exposed mice, it was shown that Nr4a1 is induced in response to β-adrenergic signaling. Nr4a1 was shown to directly bind to the promoter of Ucp3 and induce its expression, thus defining a role for Nr4a1 as an effector of brown fat thermogenesis [176]. Further studies demonstrated that Nr4a3 also binds to the Ucp1 promoter and can induce its expression [178]. Together, these data demonstrate a connection between the Nr4a’s, mitochondrial genesis and uncoupling in brown adipose tissue.

#### 4.3.4. β-Cells

Loss of Nr4a1 or Nr4a3 but not Nr4a2, in 832/13 INS-1 β-cells results in impaired mitochondrial respiration [177]. Impaired mitochondrial respiration was observed with Nr4a1 or Nr4a3 deficiency but not Nr4a2 deficiency. Impaired respiration was observed in the Leak, OxPhos and ETS states. Measurements of mRNA and protein demonstrated significant decreases in the TCA and ETC components Idh3g and Sdhb. This resulted in decreased NADH and FADH2 cycling, decreased ATP levels and ultimately decreased glucose stimulated insulin secretion [177]. These data clearly demonstrate that Nr4a1 and Nr4a3 are essential for β-cell mitochondrial respiration.

#### 4.3.5. Immune System

T-cell activation in the absence of Nr4a1 results in increased expression of the ETC components Atp5c1, Atp5f1, Atp5h, Cox6a1, Cox6b2, Cox7b, Ndufa6, Ndufa5, Ndufa8 and Ndufa11 [180]. There were also increased expression of the TCA components Dlst and Idh3a. These Nr4a1 deficient T cells had significantly greater basal and maximal respiration. Loss of Nr4a1 in macrophages impairs mitochondrial respiration in the presence or absence of LPS [181]. This corresponds with decreased mitochondrial numbers in the unstimulated state. Nr4a1 knock out macrophages have increased expression of Idh2, Idh3b and Idh3g in the stimulated state, corresponding with significantly elevated succinate levels. The increased succinate levels (and other TCA organic acids) provide added substrates for enhanced cytokine and nitric oxide production that results in enhanced atherosclerotic lesions [181] Woronicz, J.D.; Calnan, B.; Ngo, V.; Winoto, A. Requirement for the orphan steroid receptor Nur77 in apoptosis of T-cell hybridomas. *Nature* **1994**, *367*, 277–281, doi:10.1038/367277a0.

## 5. Concluding Remarks

The Nr4a family of orphan nuclear receptors play critical roles in cellular proliferation, apoptosis and fuel utilization. This occurs in a tissue specific mechanism, with a varied response depending on the tissue of interest. These data demonstrate a tissue specific ability of the Nr4a’s to either enhance proliferation or induce apoptosis. These changes impinge on the energy status of the cell, specifically mitochondrial function. This connection to replication, survival and fuel utilization places the Nr4a’s as a critical pharmaceutical target to treat various human disorders.

The mechanisms of Nr4a action are primarily through transcriptional induction or inhibition. The results acquired using the full body Nr4a1 knock out should be validated, however, using the floxed Nr4a1 knock out mice. As mentioned previously, this model results in complete Nr4a1 deletion, rather than expression of the NTD-Nr4a1 variant. These two models, used in tandem, will allow the genomic and non-genomic Nr4a1 functions to be independently defined.

It is interesting that Nr4a’s play dual roles with the mitochondria. Nr4a’s are essential for mitochondrial biogenesis and enzymatic components of the TCA cycle and electron transport chain, which impinge on fuel utilization. Conversely, Nr4a migration to the mitochondria has the ability to stabilize the mitochondria and inhibit the intrinsic apoptotic pathway. The connection between these two pathways warrants more study.

Future work needs to completely curate the Nr4a transcriptional targets through RNA-seq and ChIP-seq studies. Given the tissue specific differences, this is of high priority. Furthermore, while no endogenous ligands are yet defined, recent studies have begun to define putative endogenous ligands, as well as pharmaceutical agonists and antagonists. Greater exploration into the ability of these to modulate Nr4a function needs to be studies and their effects on various tissues. Finally, PTMs are known to modulate Nr4a family member activity. Greater definition of the types and locations of these PTMs, as well as their effect on proliferation, apoptosis and fuel utilization is needed.

## Figures and Tables

**Table 1:** Genes regulated by the Nr4a’s that are associated with cellular proliferation, defined by tissue of interest. References are given for each gene.

Tissue/Cell Line	Nr4a1	Nr4a2	Nr4a3
**Liver**			
Primary Hepatocytes (Partial Hepatectomy)	NF-κB [26]; STAT3 [26]; Cyclin B1 [26]; Cyclin d1 [26]; Cyclin E1 [26]; Cdk4 [26]; Cdk2 [26]		Cyclin D1 [27]; Cyclin E1 [27]; VCAM1 [27]; PCNA; [27]
Hepatic Stellate Cells		ERK1/2 [28,29]; p38 [28,29]; JNK [28]	
**Muscle**			
Primary Vascular Smooth Muscle	STAT3 [30]; Pim-1 [30]; NFAT [30]; Cyclin D1 [30,31]; PCNA [30,31]; p27Kip1 [32,33]	p27Kip1 [34]	Cyclin D1 [35,36,37]; Cyclin D2 [35]; PCNA [37];
Primary Ventricularmyocytes		ERK1/2 [38]; AKT [38]; DUSP2 [38]; DUSP14 [38]	
**Β-cell**			
Ins-1 832/13 Cells	Cyclin E1 [22]; E2F1 [22]; Ube2c [22]; Cdk5r1 [39]; p21Cip1 [22]; pRB [39]		Cyclin E1 [22]; E2F1 [22]; Ube’2c [22]; Cdk5r1 [39]; p21Cip1 [22]; pRB [39]
**Immune**			
Primary T Cells	Irf4 [40]		
Primary Dendritic Cells	NF-κB [41]		
Macrophage; Dendritic Progenitor Cells			RUNX1 [42]
**Cancer**			
Lung Cancer - H157 Cells		Cyclin D1 [43]	
Cervical Cancer - HeLa Cells	Cyclin D2 [44]; E2F1 [44]		
Breast Cancer - MDA-MB-231 Cells	JNK1 [45]; c-Jun [45]; Cyclin D1 [45]		
Acute Myeloid Leukemia–Mouse Models	c-Jun [18]; JunB [18]; CEBPα [46]; myc [46]; STAT1 [46]; IL-6 [46]; ERK1/2 [46]; PKB/AKT [46]	CEBPα [46]; myc [46]; STAT1 [46]; IL-6 [46]; ERK1/2 [46]; PKB/AKT [46]	c-Jun [18]; JunB [18]; CEBPα [46]; myc [46]; STAT1 [46]; IL-6 [46]; ERK1/2 [46]; PKB/AKT [46]
Acute Myeloid Leukemia–Kusami-1 Cells	c-Myc [47,48]; Bcl2 [47,48]; CBFA2T3 [47]; CSF1R [47]; PU.1 [47]; TGF-B [48]; p57 [48]		Myc [48]; Bcl2 [48]; TGF-B [48]; p57 [48]
**Endothelium**			
Primary Human Umbilical Vein Endothelial Cells	Cyclin A [49]; Cyclin D1 [49]; PCNA [49]; E2F [49]		
**Intestine**			
Intestinal Epithelium - IEC-6 Cells	p21Cip1 [50]		
**Uterus**			
Leiomyoma Smooth Muscle Cells	TGFβ3 [51]; SMAD3 [51]; collagen genes 1A1, 6A1, 6A2, and 16A1 [51]	TGFβ3 [51]; SMAD3 [51]; collagen genes 1A1, 6A1, 6A2, and 16A1 [51]	TGFβ3 [51]; SMAD3 [51]; collagen genes 1A1, 6A1, 6A2, and 16A1 [51]

**Table 2:** Genes regulated by the Nr4a’s that are associated with cellular apoptosis, defined by tissue of interest. References are given for each gene.

Tissue/Cell Line	Nr4a1	Nr4a2	Nr4a3
**Liver**			
HepG2	DNA-PKcs [93]	ERK1/2 [29]; p38 [29]; Ku80 [93]	
**Muscle**			
Primary Vascular Smooth			cIAP2 [94]
**Β-cell**			
Min6	Survivin [95]; CHOP [95]		
Primary Mouse Islets	WT1 [96]; BCL-2 [96]; SOD1 [96]		
**Immune**			
T Cells	Fasl [97]		
**Cancer**			
Pancreas Cancer – Panc-1, L.3.6pL	TXNDC5 [157		
C57BL/6 Nude mice	TRAIL [64]; PDCD1 [64]		
Colon	TRAIL [64]; PDCD1 [64]		
**Brain**			
Neurons		Adcyap1 [98]; Sod1 [98]; C-flar [98]	
**Umbilical Cord**			
HUC-F2		Caspace3 [99]; Caspase8 [99]	
**Kidney**			
Hek293	TRAPγ [100]; Hif1-α [101]; pVHL [101]	Hif1-α [101]; pVHL [101]	PARP-1 [102]
**Bone**			
U2OS		Poly-ADP-Ribose [103]	

**Table 3:** Genes regulated by the Nr4a’s that are associated with fuel utilization, defined by tissue of interest. References are given for each gene.

Tissue/Cell Lines	Nr4a1	Nr4a2	Nr4a3
**Liver**			
Primary Hepatocytes	Fbp1 [20,158]; Bpgm [20]; Pgk1 [20]; Eno3 [20,158,159]; G6pc [20,158,159]; Fbp2 [158]; Glut 2 [158]; Pgc1a [20]; Gyk [20]; PEPCK [159]; PC [159]; Lpl [20]; Ehhadh [20]; SREBP1c [20,160]; FAS [20,160]; Gpam [160]; G6Pase [20]; Lipin1 [20]; Pdk4 [20]	Fbp1 [158]; Eno3 [158,159]; G6pc [158,159]; Fbp2 [158]; Glut 2 [158]; PEPCK [159]; PC [159];	Fbp1 [158]; Eno3 [158,159]; G6pc [158,159]; Fbp2 [158]; Glut 2 [158]; PEPCK [159]; PC [159];
HepG2	G6pc [161]; PEPCK [161]; CPT1a [162];	CPT1a [162]	
**Muscle**			
Primary Muscle	Glut4 [20,163,164,165]; Pfkm [163]; Pygm [167, Eno3 [20,163,166,167,168],Bpgm [166,167]; Pgk1 [166,167]; CD36 [164]; AdipoR2 [164]; UCP2 [164]; UCP3 [164]; NADH-TR [165]; Cox1 [165]; Sdhb [165]		HK1 [169]; Pfk-1 [169]; GAPDH [169]; Pgam2 [169]; Eno3 [169]; Pkm [169],MDH [169]; AST [169]; Pdh [169]; Glut4 [169]; HK2 [169]; Stbd1 [169]; Gys1 [169]; PPP1R1A [169]; CRAT [169]; ACSL [169]; ACDH [169]; ECH [169]; 3-KCT [169]; Idh3 [169]; Ogdh [169]; Scs [169]; Sdhb [169]; Fh [169]; Mdh [169]; Atp5a1 [170]; Uqcr2 [170]; Cox1 [170]; Sdhb [170]; and Ndufb8 [170]
Cadiomyocyte	GFPT2 [171]		
C2C12	Glut4 [172]; Pfkm [172]; Pygm [172]; Eno3 [172]; Aldo1 [172]; PHKG1 [172]; PGAM2 [172]		PGC-1a [21]; PGC-1b [21]; Lipin1a [21]; Pdp1c [21]; Pdp1r [21]; Ucp3 [168];
**Adipose**			
3T3-L1	SREBP1c [173]; PPARg [173]		Glut4 [174]
HIB-1B	Ucp1 [175,176]		Ucp1 [175,176];
**β-cell**			
Ins-1 832/13	Eno1 [177,178]; Eno3 [178]; GAPDH [178]; Pgk [178]; Idh3g [177]; Sdhb [177]		Eno1 [177]; Idh3g [177]; Sdhb [177];
MIN-6	Eno3 [179]		Eno3 [179]
**Immune**			
Primary T Cells	Hk2 [180]; Aldoa [180]; Aldoc [180]; Gbe1 [180]; Agl [180]; Atp5c1 [180]; Atp5f1 [180]; Atp5h [180]; Cox6a1 [180]; Cox6b2 [180]; Cox7b [180]; Ndufa6 [180]; Ndufa5 [180]; Ndufa8 [180]; Ndufa11 [180]; Dlst [180]; Idh3a [180]		
Macrophages	Idh2 [181]; Idh3b [181]; Idh3g [181];		
Bone Marrow	Glut1 [52]; Pdk1 [52]		
**Cancer**			
AML-NB4	Glut1 [182]; Ldha [182];		
AML-THP1	Glut1 [182]; Ldha [182];		
AML-Kasumi1	Eno3 [47];		
Melanoma-MV3	TPb [183]		
Colorectal-LS-174T		Acox [184]; Cpt1m [184]; Fabp2 [184]; Fabp4 [184]	

## Abbreviations

3-KCT3	ketoacyl-CoA thiolase
5-HT	5-hydrovytryptamine
ACDH	Acyl-CoA dehydrogenase
Acox	acyl-CoA oxidase 1
ACSL	Long-chain-fatty-acid—CoA ligase
Adcyap1	Adenylate Cyclase Activating Polypeptide 1
AdipoR2	Adiponectin Receptor 2
AF-2	Activation function 2
Agl	amylo-1, 6-glucosidase, 4-alpha-glucanotransferase
AKT	Protein kinase B
Aldoa	Aldolase A
Aldoc	Aldolase C
Alo1	D-arabinono-1,4-lactone oxidase
ALP	Alprostadil
AML	Acute Myeloid Leukemia
ApoA5	apolipoprotein A5
ATP	Adenosine triphosphate
Atp5a1	ATP synthase F1 subunit alpha
Atp5c1	ATP Synthase F1 Subunit Gamma
Atp5f1	ATP synthase subunit b
Atp5h	ATP Synthase Subunit D
Bcl-2	B-cell lymphoma 2
Bpgm	Bisphosphoglycerate Mutase
BrdU	Bromodeoxyuridine
cAMP	Cyclic adenosine monophosphate
CAR	chimeric antigen receptors
CBFA2T3	CBFA2/RUNX1 Partner Transcriptional Co-Repressor 3
CD36	Cluster of differentiation 36
CD8	Cluster of differentiation 8
C-DIM12	1,1-bis(3’-indolyl)-1-(p-chlorophenyl) methane
Cdk2	Cyclin-dependent kinase 2
Cdk4	Cyclin-dependent kinase 4
Cdk5r1	Cyclin-dependent kinase 5 activator 1
CEBPα	CCAAT Enhancer Binding Protein Alpha
C-flar	CASP8 And FADD Like Apoptosis Regulator
ChIP	Chromatin immunoprecipitation
CHOP	C/EBP homologous protein
cIAP2	Baculoviral IAP repeat-containing protein3
CLACOPD	Conjugated linoleic acidChronic Obstructive Pulmonary Disease
Cox1	Cyclooxygenase 1
Cox6a1	Cytochrome C Oxidase Subunit 6A1
Cox6b2	Cytochrome C Oxidase Subunit 6B2
Cox7b	Cytochrome C Oxidase Subunit 7B
CPT1a	carnitine palmitoyltransferase 1A
Cpt1m	Carnitine Palmitoyltransferase 1B
CRAT	Carnitine O-Acetyltransferase
CSF1R	Colony Stimulating Factor 1 Receptor
CSMF	Chondrosarcoma, Extraskeletal Myxoid, Fused To EWS
Csn-b	Cytosporone B
Dlst	Dihydrolipolylysine-residue succinyltransferase component of 2-oxoglutarate dehydrogenase complex
DBD	DNA binding domain
DHE	dihydroergotamine
DIM-C-pPhCN	1,1-bis(3’-indolyl)-1-(p-cyanophenyl) methane Pin1
DIM-C-pPhCO2Me	1,1-bis(3′-indolyl)-1-(p-carboxymethylphenyl) methane
DIM-C-pPhOCH3	1,1-bis(3′-indolyl)-1-(p-anisyl) methane
DIM-C-pPhOH	1,1-Bis(3’-indolyl)-1-(p-hydroxyphenyl) methane
DNA	deoxyribonucleic acid
DNA-Pkcs	DNA-dependent protein kinase
DR5	Death receptor 5
DUSP14	Dual specificity protein phosphatase 14
DUSP2	Dual specificity protein phosphatase 2
E2F	E2 family of transcription factors
E2F1	E2 transcription factor 1
ECH	Enoyl-CoA hydratase
EDL	ETS-domain lacking
Ehhadh	Enoyl-CoA Hydratase And 3-Hydroxyacyl CoA Dehydrogenase
EMSA	Electrophoretic mobility shift assay
Eno1	Enolase 1
Eno3	Enolase 3
ER	Endoplasmic reticulum
ERK1/2	Extracellular signal-regulated kinases
ETC	Electron transport Chain
Fabp2	Fatty Acid Binding Protein 2
Fabp4	Fatty Acid Binding Protein 4
FADH2	Flavin adenine dinucleotide
FAS	apoptosis antigen 1
FASL	Fas ligand
Fbp1	Fructose-Bisphosphatase 1
Fbp2	Fructose-Bisphosphatase 2
Fh	Fumarate hydratase
G6Pase	Glucose 6-phosphatase
G6pc	Glucose-6-Phosphatase Catalytic Subunit
GAPDH	Glyceraldehyde 3-phosphate dehydrogenase
GATA2	GATA-binding factor 2
Gbe1	1,4-Alpha-Glucan Branching Enzyme 1
GDNF	Glial cell line-derived neurotrophic factor
GFP	Green fluorescent protein
Glut1	Glucose transporter 1
Glut2	Glucose transporter 2
Glut4	Glucose transporter 4
Gpam	Glycerol-3-Phosphate Acyltransferase
Gyk	Glycerol kinase
Gys1	Glycogen synthase 1
HDL	High-density lipoproteins
Hif1-α	Hypoxia Inducible Factor 1 Subunit Alpha
HK1	Hexokinase 1
HK2	Hexokinase 2
HSC	Hepatic stellate cells
HUC-F2	Human umbilical cord fibroblast
H2O2	Hydrogen peroxide
IAP	inhibitor of apoptosis
Idh3	Isocitrate dehydrogenase (NAD(+)) 3 catalytic subunit
Idh3a	Isocitrate dehydrogenase (NAD(+)) 3 catalytic subunit
Idh3g	Isocitrate Dehydrogenase (NAD(+)) 3 Gamma
IL-1β	Interleukin 1 Beta
IL-6	Interleukin 6
Irf4	Interferon regulatory factor 4
JNK	c-Jun N-terminal kinases
JNK1	c-Ju N-terminal kinase 1
JunB	Transcription Factor Jun-B
Ki67	Antigen Ki-67
Ku-80	X-Ray Repair Cross Complementing 5
Ldha	Lactate dehydrogenase A
LDL	Low-density lipids
Lipin1	Phosphatidate Phosphatase LPIN1
Lpl	Lipoprotein lipase
LPS	lipopolysaccharide
MAPK	Mitogen-activated protein kinase
Mdh	Malate dehydrogenase
MIN6	Mouse Insulinoma 6
MINOR	Mitogen-Induced Nuclear Orphan Receptor
mRNA	Messenger RNA
MTT	(3-(4, 5-dimethylthiazolyl-2)-2,5-diphenyltetrazolium bromide
Myc	MYC Proto-Oncogene, BHLH Transcription Factor
NAD+	nicotinamide adenine dinucleotide
NADH	nicotinamide adenine dinucleotide
NADH-TR	nicotinamide adenine dinucleotide tetrazolium reductase
NBRE	Nerve growth factor IB response element
Ndufa11	NADH:Ubiquinone Oxidoreductase Subunit A11
Ndufa5	NADH:Ubiquinone Oxidoreductase Subunit A5
Ndufa6	NADH:Ubiquinone Oxidoreductase Subunit A6
Ndufa8	NADH:Ubiquinone Oxidoreductase Subunit A8
NF-κB	Nuclear factor kappa-light-chain-enhancer of activated B cells
NGFI-B	Nuclear Receptor Subfamily 4 Group A Member 1
NOR1	Nuclear Receptor Subfamily 4 Group A Member 3
NOT	Nuclear Receptor Subfamily 4 Group A Member 2
Nr4a1	Nuclear Receptor Subfamily 4 Group A Member 1
Nr4a2	Nuclear Receptor Subfamily 4 Group A Member 2
Nr4a3	Nuclear Receptor Subfamily 4 Group A Member 3
NTD	N-Terminal Domain
Nur77	Nuclear Receptor Subfamily 4 Group A Member 1
NuRE	NGFIB Response Element-AAAGGTCA
Nurr1	Nuclear Receptor Subfamily 4 Group A Member 2
Ogdh	Oxoglutarate Dehydrogenase
O-GlcNAcylation	O-Linked β-N-acetylglucosamineylation
OxPhox	Oxidative Phosphorylation
p21	Cyclin-dependent kinase inhibitor 1
p27	Cyclin-dependent kinase inhibitor 1B
p38	Mitogen-Activated Protein Kinase 14
p53	Tumor protein p53
PA	Palmitic acid
PARP-1	Poly(ADP-Ribose) Polymerase 1
PC	Pyruvate carboxylase
PCNA	Proliferating cell nuclear antigen
PDCD1	Programmed Cell Death 1
PDGF	Platelet-derived growth factor
Pdh	Pyruvate Dehydrogenase E1 Alpha 1 Subunit
Pdk1	Pyruvate Dehydrogenase Kinase 1
Pdp1c	Pyruvate Dehydrogenase Phosphatase Catalytic Subunit 2
Pdp1r	Pyruvate Dehydrogenase Phosphatase Catalytic Subunit 1
PEPCK	Phosphoenolpyruvate Carboxykinase
Pfk-1	Phosphofructokinase 1
Pim-1	Proto-Oncogene, Serine/Threonine Kinase
PKB	Protein kinase B
PU.1	Hematopoietic Transcription Factor PU.1
GADPH	Glyceraldehyde-3-Phosphate Dehydrogenase
PGAM	Phosphoglycerate Mutase 2
PGC-1α	PPARG Coactivator 1 Alpha
PGC-1β	PPARG Coactivator 1 beta
Pgc1a	PPARG Coactivator 1 Alpha
Pgk1	Phosphoglycerate Kinase 1
PHKG1	Phosphorylase Kinase Catalytic Subunit Gamma 1
Pin1	Peptidylprolyl Cis/Trans Isomerase, NIMA-Interacting 1
Pkm	Pyruvate Kinase M1/2
PPARγ	Peroxisome Proliferator Activated Receptor Gamma
PPP1R1A	Protein Phosphatase 1 Regulatory Inhibitor Subunit 1A
pRB	Retinoblastoma protein
PTM	Post-translational modifications
PTMs	Post-translational modifications
pVHL	von Hippel–Lindau tumor suppressor
Pygm	Glycogen Phosphorylase, Muscle Associated
RNA	Ribonucleic acid
ROS	Reactive oxygen species
RUNX1	Runt-related transcription factor 1
Scs	Succinate-CoA Ligase ADP-Forming Beta Subunit
Sdhb	Succinate Dehydrogenase Complex Iron Sulfur Subunit B
shRNA	small hairpin RNA
siRNA	small interfering RNA
SMAD3	Mothers against decapentaplegic homolog 3
SOD1	Superoxide dismutase
SREBP1c	Sterol Regulatory Element Binding Transcription Factor 1
STAT3	Signal transducer and activator of transcription 3
Stbd1	Starch Binding Domain 1
STZ	streptozotocin
TCA	Tricarboxylic acid cycle
TG	Thapsigargin
TGF-β1	Transforming growth factor beta-1
TGFβ3	Transforming growth factor beta-3
TINUR	Transcriptionally-Inducible Nuclear Receptor
TNF	Tumor Necrosis Factor
TPβ	Hydroxyacyl-CoA Dehydrogenase Trifunctional Multienzyme Complex Subunit Beta
TRAIL	TNF-related apoptosis-inducing ligand
TRAPγ	TRAP-Complex Gamma Subunit
Treg	Regulatory T Cell
TUNEL	Terminal deoxynucleotidyl transferase-mediated dUTP nick-end labeling
TXNDC5	Thioredoxin Domain-Containing Protein 5
Ube2c	Ubiquitin-conjugating enzyme E2 C
Ucp1	Uncoupling Protein 1
UCP2	Uncoupling Protein 2
UCP3	Uncoupling Protein 3
Uqcr2	Ubiquinol-Cytochrome C Reductase Core Protein 2
VCAM1	Vascular cell adhesion protein 1
VEGF	Vascular endothelial growth factor
VEGF-A	Vascular endothelial growth factor A
VSMC	Vascular smooth muscle cell
WT1	Wilms tumor
